# Atlanta’s Potable Water

**Published:** 1883-09

**Authors:** 


### ATLANTA'S POTABLE WATER.

Continued from August Number, page 687.

We showed in the last number of the REGISTER the impure, unsafe condition of many of the wells of the city, as sources of wholesome, drinkable water—that the more centrally placed wells were already dangerously contaminated, and that this process of contamination was increasing in area and intensity *pari passu* with the growth of the city.

But were every well within the limits of the city to pour out water as pure as that which trickles from the snows of Mt. Everest, it would be a supply utterly inadequate for even the sanitary requirements of a great city. It was in view of this fact that the city fathers, a few years ago, established the water works system of South river, from which the chief water supply of Atlanta is at present drawn.

So far as it goes this supply is good, and equal to expectations; both the microscope and chemical analysis prove it to be pure from organic contamination, and when perfectly filtered is as clear, sparkling and palatable drinking water as would be desired. But the supply of water from this source falls far short of the sanitary necessities even of the city, to say nothing of the requirements of the fire department and a great and growing manufacturing interest.

No city abreast of the civilization of the times can afford to ignore for a day the claims of public sanitation; and the chief factor in all urban sanitation is pure water, a super-abundance of pure water—water everywhere. There must be no cry of economy in this The supply should be so abundant that there will be enough for every contingency—for every useful application, with a large margin for waste. The man who raises the cry of retrenchment and economy in enterprises promotive of a city's sanitation, moved by the ignorance or low aim of the scheming politician, is unfit for a public trust. It is one of the cheering signs of the times that such men, though fancying themselves too sagacious to make a misstep, are really often far below the range of public information and opinion, and being suddenly hurled over the dead-line, go down at the very moment they thought themselves most sure of success. The people of Atlanta are now ready for any scheme that will give them, at reasonable cost, an abundant and never failing supply of pure, wholesome water. The necessity of an enterprise capable of effecting this is a foregone conclusion. The city must have more and better water than she has, or cease forever to boast of her achievements, and expect only loss and declension in the future.

But the question recurs, where shall this water supply be got? We answer boldly, from the Chattahoochee. The work and expense required for the successful accomplishment of this is less than that which would rebuild the Kimball House from its ashes, while as regards general utility, public and private benefit, it is beyond all comparison more important.

The volume of water afforded by the river, taken at extreme low water mark, the mark of the drouth of 1881, at Seven Island Shoal, has been ascertained, we learn from Colonel Frobel to be 700 cubic feet per second. From tributary streams there are 100 feet per second more, making in all 800 cubic feet. A canal forty-six feet wide at the water surface, seven feet deep and with a fall of 5.10 foot per mile will furnish 500 cubic feet of water per second. The maximum evaporation from surface of such canal would be five cubic feet per second, so that we would have 500 cubic feet per second drawn from 800 cubic feet per second, and this amount taken at the low estimate of the dryest season known in Georgia. It is easy to see that this supply would not only be equal to all the requirements of domestic life in this city, but to those of sanitation and manufacturing as well.

Five hundred cubic feet of water, delivered thus every second in the city, would give in twenty-four hours 43,-200,000 cubic feet, enough to give ample motion to all the machinery and mills that could be planted along the canal, and to supply besides every man, woman and child in a population of 100,000 with 432 cubic feet of water each day of twenty-four hours, or eighteen cubic feet every hour; equal to 135 gallons per head. The surpassing advantage, however, of such a system of water supply would be the admirable facility afforded by it for the full and perfect cleansing of the city daily. The problem of an ideal system of sanitation would be solved. A tunnel sewer constructed from one of the lower levels of the canal could receive all the secondary sewerage of the city, and could be so arranged that on the shutting down of the mills at night it would be filled with the surplus water, and when the sluice gates were opened in the morning this back water would be drawn out, and with it the entire sewerage of the city. Thus every night the whole sewer system of the city could be washed out. The generation of poisonous gases would be an impossibility, and the city of Atlanta become the best drained, the cleanest and healthiest city in America.

A word as to the feasibility of this great work. The water surface at Seven Island Shoals is twenty-seven fee higher than the site of the carshed, one of the highest points in the city.

One United States and two railroad surveys, by accomplished engineers, among them Frobel and Grant, have settled this question beyond any reasonable doubt. Sixty-five miles must be the length of the canal, but encumbered throughout its entire course with not a single aqueduct nor lock, and with only one short tunnel. This single tunnel will be under a railroad track to obviate the necessity of a viaduct.

The construction of such a canal for the purpose of procuring pure, palatable water, and for purposes of public sanitation alone, cannot be much longer delayed, awaiting the tardy decisions of capitalists at home, for already the water supply from South river is inadequate and the advancing population of the city is rapidly peopleing the very watershed, whose intact condition has heretofore been the only guarantee of the continued purity of the South river water. The logic of facts, faster than the sagacity of man, is hastening forward the inception of this grandest of Atlanta's enterprises, and when it becomes an accomplished fact, her extraordinary advancement in population and in all the improvements that make up a city's prosperity, will once more recall the memorable prophecy of Mr. Stephens, and the year 1901 doubtless witness the anniversary of the canal's completion by a population of 200,000 people hardby the waters of the Chattahoochee.

The long cherished possibility of Atlanta becoming a great manufacturing centre alone necessitates the construction of this canal. It presents a splendid financial opportunity which is attracting, at this moment, the attention even of foreign capitalists. Not two hours ago we were shown a batch of letters just received from certain

English capitalists, who are earnestly negotiating for the privilege of undertaking the work. The irrepressible conflict is between steam and water power. But in the matter of economy steam power can never compete successfully with the cheaper power of water. Experiment has settled this point forever all over Georgia if not for the entire South. It is well known that factories run by steam in this State have failed to be remunerative, except here and there one, which being very favorably situated as respects railroads and navigable waters, it has enjoyed a monopoly of transportation. The Atlanta Cotton Factory is not one of those exceptions. To further the cause of this enterprise it was claimed, and published to the world that steam power is really cheaper than that of water. The necessary capital was raised, the building erected, and filled with the best machinery of the kind in the world. It went to work apparently under the most favorable auspices. Alas! A subsequent troublous history tells all the story of its experience with steam as an economical motive power. Under very favorable conditions the difference of cost between steam and water power is six dollars per day for every 1,000 spindles. The Atlanta cotton factory has, we believe, 10,000 spindles; on that estimate, therefore, it would save about sixty dollars a day, or more than $18,000 annually by using water instead of steam.

The Chattahoochee canal will bring to the city a mechanical energy of not less than 30,000 horse power sufficient to propel at least two hundred such mills as the Atlanta cotton factory, running two million spindles. The reader can readily estimate for himself the immense economical advantages of such a work to the city, on the basis of the above facts.

But steam power in Atlanta is as far from being applied under very favorable conditions, as the transportation of its products is from the advantages of a monopoly. Hence again, the financial results founded on the given difference of cost between steam and water power, must be largely increased.

A few words must be added in regard to the purity of the Chattahoochee water. This is remarkable both by chemical analysis and by the test of experience.

The Chattahoochee belongs to the same system of water drainage as its twin sister, the beautiful Savannah. They have their origin in the same mountain range and geological formation, the granite and freestone of the Blue Ridge. They emerge, as it were, from the lap of the same mountain valley, so that whatever may be claimed for the waters of the one the same is true of those of the other.

The Savannah has been long in high repute for the purity and sweetness of its drinkable water. The sailors plying their trade along the east coast of the United States have time out of mind regarded Savannah river water as the best with which to supply their vessels for a voyage, and seldom does a passing ship lose the opportunity of replenishing its water casks from the mouth of that river. Could England have such a stream as the Chattahoochee running within nine miles of its great overgrown capital, soon every precious drop of its water would be treasured for distribution throughout its streets and houses and to the joyous people no mere money standard could ever express the real value of the boon. Every stream in England is polluted.

